# COVID-19 Mortality Underreporting in Brazil: Analysis of Data From Government Internet Portals

**DOI:** 10.2196/21413

**Published:** 2020-08-18

**Authors:** Lena Veiga e Silva, Maria Da Penha de Andrade Abi Harb, Aurea Milene Teixeira Barbosa dos Santos, Carlos André de Mattos Teixeira, Vitor Hugo Macedo Gomes, Evelin Helena Silva Cardoso, Marcelino S da Silva, N L Vijaykumar, Solon Venâncio Carvalho, André Ponce de Leon Ferreira de Carvalho, Carlos Renato Lisboa Frances

**Affiliations:** 1 Federal University of Pará Belém Brazil; 2 University of Amazon Belém Brazil; 3 Federal University of São Paulo São José dos Campos Brazil; 4 National Institute for Space Research São José dos Campos Brazil; 5 University of São Paulo São Carlos Brazil

**Keywords:** Brazil, COVID-19, mortality, underreporting, respiratory system diseases, public health, pandemic, time series, forecasting

## Abstract

**Background:**

In Brazil, a substantial number of coronavirus disease (COVID-19) cases and deaths have been reported. It has become the second most affected country worldwide, as of June 9, 2020. Official Brazilian government sources present contradictory data on the impact of the disease; thus, it is possible that the actual number of infected individuals and deaths in Brazil is far larger than those officially reported. It is very likely that the actual spread of the disease has been underestimated.

**Objective:**

This study investigates the underreporting of cases and deaths related to COVID-19 in the most affected cities in Brazil, based on public data available from official Brazilian government internet portals, to identify the actual impact of the pandemic.

**Methods:**

We used data from historical deaths due to respiratory problems and other natural causes from two public portals: DATASUS (Department of Informatics of the Unified Healthcare System) (2010-2018) and the Brazilian Transparency Portal of Civil Registry (2019-2020). These data were used to build time-series models (modular regressions) to predict the expected mortality patterns for 2020. The forecasts were used to estimate the possible number of deaths that were incorrectly registered during the pandemic and posted on government internet portals in the most affected cities in the country.

**Results:**

Our model found a significant difference between the real and expected values. The number of deaths due to severe acute respiratory syndrome (SARS) was considerably higher in all cities, with increases between 493% and 5820%. This sudden increase may be associated with errors in reporting. An average underreporting of 40.68% (range 25.9%-62.7%) is estimated for COVID-19–related deaths.

**Conclusions:**

The significant rates of underreporting of deaths analyzed in our study demonstrate that officially released numbers are much lower than actual numbers, making it impossible for the authorities to implement a more effective pandemic response. Based on analyses carried out using different fatality rates, it can be inferred that Brazil’s epidemic is worsening, and the actual number of infectees could already be between 1 to 5.4 million.

## Introduction

### Background

On December 31, 2019, the World Health Organization (WHO) received a report from China about cases of pneumonia of unknown etiology in Wuhan, Hubei Province. By January 7, 2020, Chinese scientists isolated the virus, identifying it as a novel coronavirus and initially referred to it as 2019-nCoV (later named severe acute respiratory syndrome coronavirus 2 [SARS-CoV-2]) [1-3]. The virus, which causes coronavirus disease (COVID-19) [4], ended up spreading to other countries and, by late January 2020, the WHO declared it an Public Health Emergency of International Concern; the outbreak was declared a pandemic on March 11, 2020.

The global impact of the virus has been of great concern and has overburdened public health systems worldwide. It can be considered the first true global epidemic of this magnitude in the digital era [5]. COVID-19 is an acute respiratory disease, often severe, which may become fatal to those who are infected [1]. The disease occurs when one comes into contact with contaminated secretions, in particular, large respiratory droplets, as well as when in contact with contaminated surfaces [3]. It disseminates rapidly, compromising the health of a large number of people, and consequently overwhelms health care infrastructure and resources. Decision makers must act immediately to minimize the effects of the disease and flatten the contagion curve to control both spread and fatalities.

As the disease propagates, the burden to health care systems increases, despite a large number of asymptomatic cases. Studies in China show that 62% of COVID-19 transmissions occur as a result of asymptomatic and presymptomatic individuals [6]. Thus, there is a high chance that the actual number of infectees is far larger than that officially announced. Moreover, it is very likely that the actual proliferation of the disease is being underestimated, with a very high number of underreported cases.

### The Pandemic in Brazil

Outside the Asian continent, the disease was initially concentrated in Western Europe and North America. In a short period of time, however, it expanded to other parts of the world like Africa and Latin America [7]. Brazil’s first case and death were announced on February 26th and March 17th, respectively. Since then, the disease has been spreading rapidly, devastating almost all regions of the country; at present, Brazil has the fourth highest number of deaths and the second highest number of confirmed infections [8]. According to the coronavirus website of Brazilian Ministry of Health [9], there were more than 700,000 confirmed cases and almost 40,000 deaths, as of June 9, 2020.

The country’s difficult situation is magnified due to social inequalities. According to the Brazilian Institute of Geography and Statistics (IBGE) [10], Brazil has a population of approximately 204.5 million people, of which 85% are <59 years of age. The country has 65 million (31.8%) people living in poor or extreme conditions of poverty (eg, precarious living, lack of basic sanitation, reduced access to health care, etc). It has recorded an unemployment rate of 12.2% in the first quarter of 2020 [10]. Public measures tailored to these populations are necessary. On a positive note, Brazil has a government-funded Unified Healthcare System (Sistema Único de Saúde, SUS) that is responsible for 70% of the population [11].

Brazil has 27 states divided territorially into five major regions: North, Northeast, Midwest, Southeast, and South, with specific climatic, social, and economic characteristics. According to the IBGE [12], the North region has the lowest demographic density, with 4.72 inhabitants/km^2^ and a Human Development Index (HDI) of 0.683. The Southeast region is more developed and the most populous, with approximately 92 inhabitants/km^2^, and accounts for 55.2% of the national gross domestic product (GDP) (HDI=0.784). There is greater social inequality in the Northeast region (HDI=0.608).

A proper estimation of underreported or wrongly reported cases is necessary for a better understanding of the actual epidemic scenario; this will allow for necessary and effective measures to be undertaken by the authorities. In Brazil, underreporting is due to the low rate of testing per 1 million inhabitants. Additionally, there is significant delay in the reporting of test results [13]. During the first weeks of the COVID-19 outbreak, Brazil had tested all suspected cases as well as those that had been in contact with a confirmed case. However, low availability of RT-PCR (reverse transcription polymerase chain reaction) tests forced the Ministry of Health to recommend testing for only serious cases [9]. This approach was also extended to those belonging to high-risk groups (eg, health care professionals).

Different grades of testing and reporting are observed in other countries [14] so it is difficult to understand what the actual situation in Brazil and its states looks like. According to WorldoMeter [15], 1,182,581 tests have been conducted in Brazil so far, a rate of 5566 tests per 1 million inhabitants, which is much lower than that other countries like Spain (86,921 tests per 1 million inhabitants), Portugal (78,030 tests per 1 million inhabitants), and the United States (53,156 tests per 1 million inhabitants).

This undersampling leads to a high degree of underreported cases, which affects estimates of the actual fatality rate of the disease [7]. Therefore, it is of fundamental importance to uncover the degree to which underreporting has occurred in order to define and establish public health policies related to pandemic response.

It has been suggested that the reproduction number (R) must be less than 1 in order to reduce the number of infected cases [7]. However, although several Brazilian states have adopted isolation, social distancing, and even lockdown measures, noncompliance is an issue.

### Official Brazilian Government Internet Portals

With the increasing spread of SARS-CoV-2 in Brazil, there has been a considerable growth in the population's interest for information about the disease. According to Google Trends [16], web queries for the term “Coronavirus” increased substantially in Brazil, reaching its peaks on March 15th and 21st. The most searched terms included “cases of coronavirus,” “deaths coronavirus,” “coronavirus symptoms,” and “coronavirus update.” During this period, access to news about the virus increased by more than 5000% when compared with the previous period. Additionally, tweets related to the novel coronavirus were among those that were most commented on; in Brazil, topics such as chloroquine, Minister of Health, quarantine, and treatment of coronavirus were the most sought after on Twitter [17].

To manage this increase in interest, several official internet portals were created by the Brazilian municipal, state, and federal bodies for dissemination, monitoring, and guidance. However, the data presented by these public internet portals are contradictory and inaccurate. Some of the data released highly underreport the true number of cases, leading to false perceptions that the contagion is under control.The population must trust the data provided to them in order to accept proposed recommendations [18].

We believe that by aggregating officially available information into a single internet portal, removing contradictions, and using reliable sources, we can gather support from the Brazilian populace to follow WHO-recommended guidelines, thus reducing the contagion rate in Brazil. This portal is under development as part of the work presented in this paper and will enable policy and decision makers to base their assessments on scientific evidences and guide citizens in adopting recommended measures and behaviors (eg, social distancing, frequent hand sanitizing, and more attention to hygiene issues).

### This Study

The work described in this paper conducts an investigation into underreported deaths with respect to COVID-19 based on historical mortality data due to respiratory problems and other natural causes. These data are publicly available on the internet through the two main portals of the Brazilian government: the Mortality Information System (SIM) of DATASUS (Department of Informatics of the Unified Healthcare System) [19] and the Brazilian Transparency Portal of Civil Registry [20]. The aim is to systematize the contradictory information in these portals to provide a more representative picture of the pandemic and estimate the possible number of death reports that were incorrectly recorded. These data were used to build time-series models (modular regressions) with the ability to predict the expected mortality rate for 2020. This was done to assess whether significant disagreement is present between the real and expected number of deaths for this period. By estimating the actual number of COVID-19–related deaths, it is possible to determine the number of infected people from officially published fatality rates.

In this study, we used as case studies the capital cities of three regions that were most affected by the pandemic: North (Belém and Manaus), Northeast (Fortaleza and Recife), and Southeast (São Paulo and Rio de Janeiro). The resulting mortality underreporting scenario will be considered for the entire country as these cities represent around 47% of the total deaths in Brazil as of June 9, 2020 [9].

## Methods

### Overview

**Figure 1:**
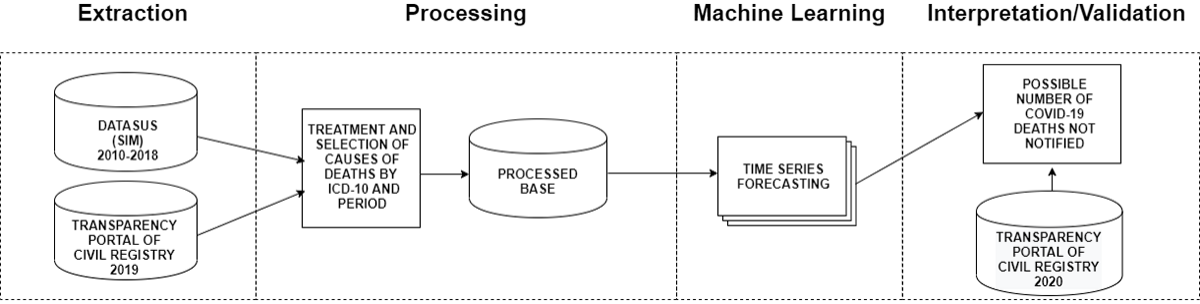
Methodology diagram adapted from Fayyad et al [21]. DATASUS: Department of Informatics of the Unified Healthcare System; SIM: Mortality Information System; ICD-10:International Statistical Classification of Diseases and Related Health Problems–10th Revision; COVID-19: coronavirus disease.

We followed the Knowledge Discovery in Databases workflow to extract new and relevant data to enable decision making (Figure 1). Two public databases with nationally consolidated data were consulted: DATASUS and the Brazilian Transparency Portal of Civil Registry. In the analysis, these steps were followed: data extraction, data processing, machine learning, and data interpretation and validation. Health care specialists aided in some of these steps.

### Data Extraction

Data were collected from two government sources accessible for public use. The registers present in both databases follow the international standards set by the WHO.

Part of the data collected for this research was extracted from DATASUS (SIM) [19]. It is a system from which one can access regular information on mortality rates in Brazil to assist public health management sectors [19]. Data were extracted for the 2010-2018 period for all capital cities of Brazilian states. It is important to clarify that SIM is updated annually; hence, 2019 was not considered since the data is not available yet. Each entry in the SIM database is highly detailed, concisely presenting all the information contained in the death certificate.

Another source was the Brazilian Transparency Portal of Civil Registry [20]. It comprises deaths registered due to COVID-19 (confirmed or suspected) and respiratory diseases, such as severe acute respiratory syndrome (SARS), pneumonia, and respiratory failure. The civil registry data website is based on death certificates sent by the registry offices countrywide for deaths that take place in hospitals, residences, public roads, etc. Data were collected for the January 1 to June 1, 2020 period, as well as the same period for the year 2019. For the years 2019 and 2020, the civil registry portal records another category—deaths from other causes (when these were unrelated to COVID-19 but related to respiratory problems). This last category was also considered in this study.

The Brazilian civil registry portal presents the data duly notarized by the civil registry offices and follows a series of legal timelines established by the Brazilian Constitution—a family has 24 hours after the death of a member to notify the registry office, and in turn the registry office has up to 5 days to duly register the death; within 8 days the Information Center of Civil Registry receives the report, which is published by the civil registry portal. Therefore, there may be a delay of 14-15 days for the portal to publish a record.

In addition to the large delay in the Transparency Portal of Civil Registry death reports, it is important to highlight that the update frequency might be different for each city. For certain regions, the delays are even longer. In general, the data for capital cities are updated more frequently. For this reason, although the data were collected on June 1st, the analysis will be conducted using data made available up to May 21st. By adopting this procedure, we can mitigate the effect of late notifications in the analysis.

### Data Processing

Data were preprocessed by removing missing and duplicated information to improve quality, so that more significant results can be presented. This removal of data was not substantial, and the entire data set was stored in a single database.

The time series of deaths due to the previously mentioned diseases were from DATASUS (SIM) and were duly processed to be concatenated with those from the Transparency Portal of Civil Registry. Following the conditions used by the civil registry portal, each occurrence of death was classified according to the International Statistical Classification of Diseases and Related Health Problems (ICD) [22] and based on the last, underlying, and immediate cause of death present in the death certificate. The fields used in the database for date of death and ICD are mandatory. The nested classification conditions are summarized in Table 1.

In order to classify each record of data from DATASUS (SIM) based on the listed conditions, it was necessary to identify the ICDs [22]. Thus, the corresponding IDs for the causes of deaths from the civil registry portal are shown in Table 2. Health care specialists contributed to identifying and classifying the ICDs.

In order to merge the databases, data referring only to death records for capital cities were extracted from DATASUS (SIM). These records were then aggregated on a daily basis. Therefore, both the databases are now compatible with respect to their indices and columns, making it possible to concatenate the data and merge into a single data set, which was then used to conduct this study.

**Table 1:** Conditions established by the Transparency Portal of Civil Registry to classify deaths.

Order	Condition
1	If there is any mention of COVID-19^a^ in the death certificate, suspected or confirmed, it was considered a death attributed to COVID-19.
2	If there is any mention of severe acute respiratory syndrome (SARS), it was considered the cause of death.
3	If there is any mention of pneumonia, it was considered the cause of death.
4	If respiratory failure is listed as the only cause, it was considered the cause of death.
5	If the certificate does not mention any of the above conditions, the cause of death was considered as “other”.

^a^COVID-19: coronavirus disease.

**Table 2:** International Statistical Classification of Diseases and Related Health Problems–10th Revision (ICD-10) classification adopted by the Transparency Portal of Civil Registry.

Disease	ICD-10 classification
Severe acute respiratory syndrome (SARS)	I260, U04, J22, J100, J110
Pneumonia	J12, J13, J14, J15, J16, J180, J181, J182, J188, J189, B953, B960, B961
Respiratory failure	J96

### Time-Series Forecasting Model

The models used for time-series prediction were adjusted to predict the expected number of deaths for 2020 based on a historical series from 2010 to 2018 for six capital cities. In order to conduct the experiment, training based on the modular regression model FbProphet [23] was employed. The resulting decomposed time-series model is shown in the following equation:


y (t) = g(t) + s(t) + h(t) + ε_t_  **(1)**

where, according to the model by Harvey and Peters [24], *g(t)* represents a function of tendency used to capture nonperiodic changes in a historical series; *s(t)* refers to periodic seasonality, representing the annual, monthly, and weekly recurring behavior; and *h(t)* represents the effects of holidays on the data. The component ε*_t_* is used to represent peculiar changes not included in the model.

The main component of equation 1, *g(t)*, is used to represent the trend model. Equation 2 refers to this component when used in forecasting problems that exhibit a linear trend with change points:


g(t) = (k + a(t)Tδ)t + (m + a(t)T γ) **(2)**

where is the growth rate, δ is a vector containing adjustments to the growth rate, is used as an offset parameter, and γ is used as an adjustment vector for the parameter . The vector *a(t)* is used to define the change points, allowing the growth rate to be adjusted accordingly.

As previously mentioned, component *s(t)* of equation 1 is used to represent the seasonal influences and recurring behaviors present in the time series. Those seasonal effects rely on a Fourier series representation (equation 3). It is possible to adjust the parameter *P*, represented in days, in order to obtain the desired seasonality (eg, *P*=7 for weekly seasonality).

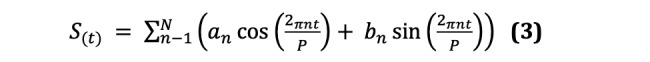



In order to fit the model to the data, the time-series forecasting is treated as a curve-fitting problem, taking the data seasonalities and holiday effects into consideration [23]. The framework uses an implementation of the Limited-memory Broyden-Fletcher-Goldfarb-Shanno algorithm, referenced by Zhu et al [25], in order to find a maximum *a posteriori* estimate.

### Data Interpretation and Validation

For this analysis, we used data on COVID-19–related deaths of the six capital cities with the highest number of deaths recorded by the civil registry website: Belém (capital of Pará), Fortaleza (capital of Ceará), Manaus (capital of Amazonas), Recife (capital of Pernambuco), Rio de Janeiro (capital of Rio de Janeiro), and São Paulo (capital of São Paulo).

Once the processing workflow and data cleaning are completed, it is possible to devise a system to predict trends in deaths caused by respiratory issues, as well as to predict the expected behavior of diseases for 2020. Based on the number of deaths per year for each disease for the capital cities under consideration, an estimate of deaths was calculated for normal conditions (ie, no pandemic). Thus, the difference between the number of expected cases for 2020 and recorded cases for 2020 was determined. Next, this extrapolation was added to the deaths reported for COVID-19, allowing us to estimate the actual number of deaths due to the pandemic. With this analysis, the actual cause of sudden increase in deaths, not only due to respiratory issues but also other deaths, could be estimated.

## Results

We conducted an exploratory analysis of the data to evaluate patterns in the number of deaths during the pandemic. Subsequently, we employed a time-series model to estimate the number of incorrectly reported figures.

### Exploratory Data Analysis

**Figure 2:**
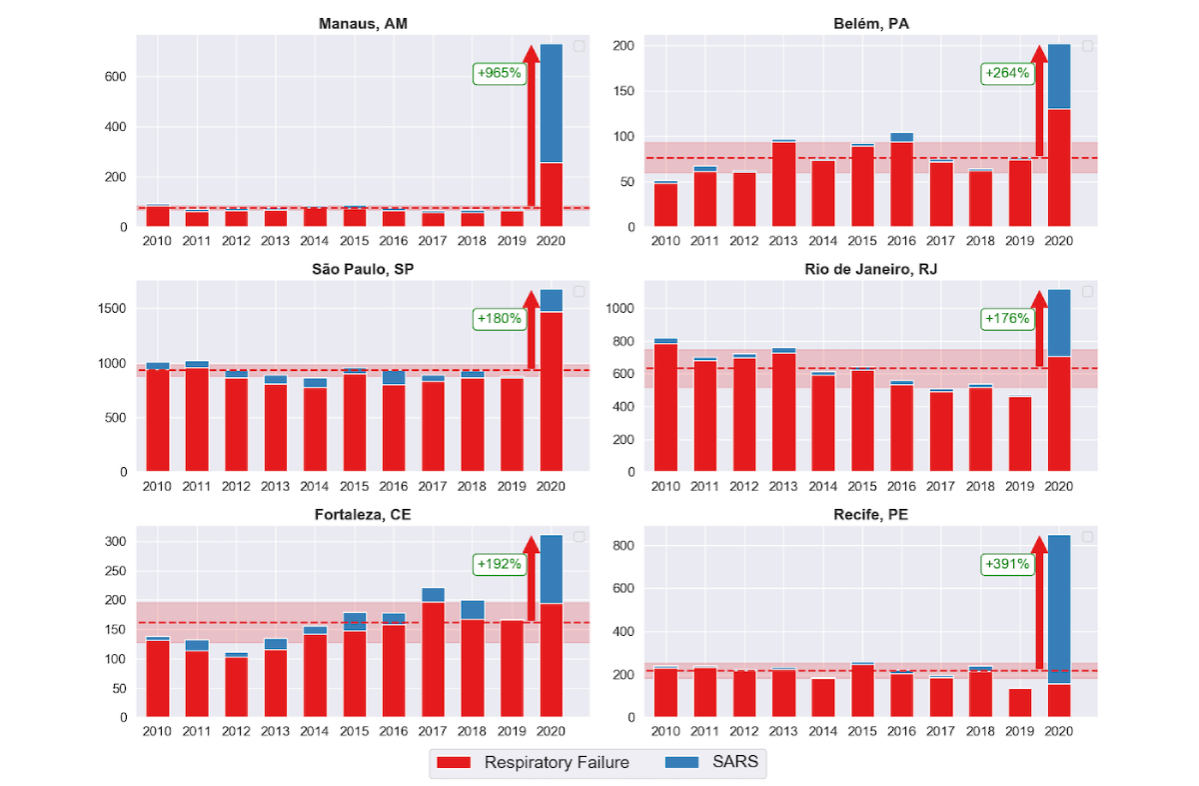
Increases in the number of deaths due to respiratory failure and severe acute respiratory syndrome (SARS).

The historical series of deaths for 2010-2018 (extracted from SIM [19]), 2019, and 2020 (extracted from civil registry portal [20]) for a same period for all the mentioned years were considered. We observed an increase of 965% (from 75.8 to 732) with respect to the average number of registered deaths due to SARS and respiratory failure per year for Manaus, one of the most affected capitals (Figure 2). Due to a high disagreement from the historical series of deaths for the mentioned period that coincides with the pandemic period, it is necessary to investigate the cause of this large difference.

Recife, Belém, Fortaleza, São Paulo, and Rio de Janeiro also presented a significant increase in the number of deaths in 2020. Figure 2 illustrates the disagreement between the number of deaths that occurred between the 13th and 19th weeks of the epidemic in 2020 with respect to the average of the historical series for the same period in previous years for both the diseases—respiratory failure and SARS—that presented a large variation. It is possible to observe distinct behaviors in the discrepancy in records for each city. In Recife, the substantial increase in SARS cases draws a great deal of attention, while Manaus presented a considerable increase for all causes of death. Despite the increase being more significant for SARS and respiratory failure, we observed occasional discrepancies in regard to pneumonia and deaths due to other causes. The mean number of deaths and standard deviations, along with the percentage of increase with respect to the average of the historical series for these diseases, are presented in Table 3.

As previously mentioned, we observed a major discrepancy for SARS-related deaths for all cities. A sudden increase of 6991% (from 9.8 to 685) for SARS in Recife, for example, might be associated with errors in reporting. SARS, first detected in China in November 2002, is caused by a type of coronavirus called severe acute respiratory syndrome coronavirus (SARS-CoV), with symptoms similar to COVID-19, causing a severe respiratory viral infection [26]. Thus, it is possible that the similarities between the diseases can compromise the accuracy of death records.

**Table 3:** Mean (SD) for the historical series and percent increase/decrease of deaths caused by respiratory failure, pneumonia, severe acute respiratory syndrome (SARS), and other causes.

City		Respiratory failure	Pneumonia	SARS	Other causes
**Belém**					
	Mean (SD)	72.7 (15.62)	180.6 (42.09)	3.6 (2.54)	491.1 (72.5)
	Increase/decrease (%)	+78	+37	+1900	–1
**Fortaleza**					
	Mean (SD)	144.3 (29.09)	442.2 (147.90)	17.9 (10.12)	1474.6 (161.92)
	Increase/decrease (%)	+35	–1	+553	–11
**Manaus**					
	Mean (SD)	66.8 (9.01)	259.4 (41.80)	9.0 (2.16)	1162.6 (122.70)
	Increase/decrease (%)	+283	+192	+5188	+69
**Recife**					
	Mean (SD)	207.5 (32.2)	307.1 (39.3)	9.8 (6.98)	1963.2 (305.40)
	Increase/decrease (%)	–24	­–43	+6991	–25
**Rio de Janeiro**					
	Mean (SD)	611.3 (108.85)	1501.1 (166.94)	22.7 (7.64)	6065.1 (495.47)
	Increase/decrease (%)	+15	+16	+1701	–5
**São Paulo**					
	Mean (SD)	861.8 (59.12)	2933.3 (247.11)	70.4 (30.82)	8418.1 (571.08)
	Increase/decrease (%)	+70	–2	+192	+6

### Time-Series Prediction

The exploratory analysis identified values that were much higher than the average of the historical series for registered deaths during the pandemic period. For this reason, in this section we further analyze the results obtained from the time-series models developed to compare the expected trend (predicted) and the actual trend.

We trained the time-series models with data from January 2010 to May 2019. The model was adjusted to individually predict the behavior of each of the three diseases and deaths over other causes in each.

To compute the error metrics, each model was initially trained using 7 years of data. A cross-validation process was then conducted for the remaining data for every 90-day cutoff at a 470-day horizon. Table 4 shows the absolute errors for the validation set predictions.

**Figure 3:**
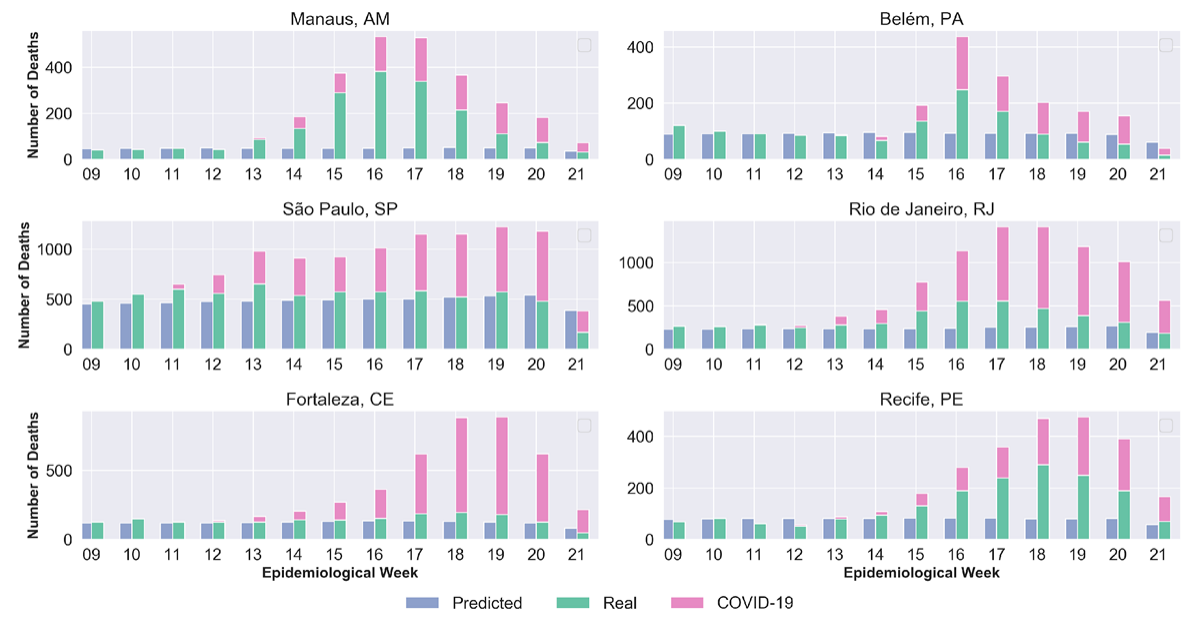
Predicted and actual deaths per epidemiological week related to respiratory diseases. COVID-19: coronavirus disease.

The models were then used to predict data up to May 21, 2020, to be compared with the actual data presenting the observed anomalies. Figure 3 compares the number of registered deaths (actual) from civil registry website, including deaths due to COVID-19, and the predicted deaths returned by the time-series models. The results are grouped by epidemiological weeks and considers data from the 9th week until the 21st week of 2020. Our results demonstrated that each city presented a different trend with respect to the peak periods for disease activity within the considered timeframe. Therefore, analysis must be performed considering their specific periods.

**Table 4:** Mean absolute error (MAE) and mean absolute percentage error (MAPE).

City		Respiratory failure	Pneumonia	SARS^a^	Other causes
**Belém**					
	MAE	1.61	2.64	0.40	5.15
	MAPE	9.6	11.6	33.5	8.3
**Fortaleza**					
	MAE	1.81	2.34	0.57	6.59
	MAPE	11.4	2.6	37.0	10.1
**Manaus**					
	MAE	0.75	1.88	0.34	4.47
	MAPE	14.0	10.0	28.4	8.3
**Recife**					
	MAE	1.91	2.34	0.59	7.45
	MAPE	12.3	7.8	40.0	6.0
**Rio de Janeiro**					
	MAE	2.77	4.99	0.50	13.38
	MAPE	6.7	6.8	25.8	5.2
**São Paulo**					
	MAE	3.34	7.78	0.96	12.27
	MAPE	2.4	3.1	36.4	2.3

^a^SARS: severe acute respiratory syndrome.

**Figure 4:**
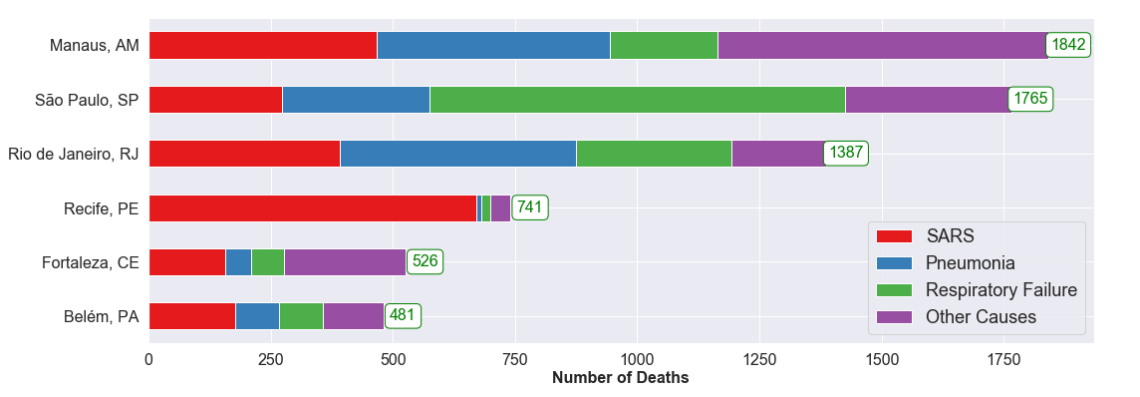
Estimated number of deaths wrongfully attributed to respiratory system diseases for the considered periods. SARS: severe acute respiratory syndrome.

Taking into account the peak periods for each city, predicted figures are smaller than the actual values in terms of the days with a high number of deaths due to respiratory and other causes. The estimates of errors in death reports for each disease, per city, are shown in Figure 4. The number at the end of each bar represents an estimate, in absolute numbers, of the number of cases that deviate from the expected pattern, and most probably were incorrectly recorded.

Each city, with its own particularities (Figure 4), has its causes of death recorded differently. Table 5 presents the considered periods for each city and the difference between the number of reported cases and the number of predicted cases both quantitatively and percentagewise. The last column shows the total difference in the number of deaths for the period not covered in the historical series.

The predicted values show different increases for the investigated cities. For São Paulo, where the first COVID-19 death confirmed by the Brazilian government occurred in the 11th week, the increase was 24.4% (from 7238 to 9004). For the other cities, the following increases were observed: 144.7% (from 1274 to 3117) for Manaus, 128.9% (from 575 to 1317) for Recife, 99.6% (from 485 to 968) for Belém, 41.2% (from 1279 to 1806) for Fortaleza, and 39.9% (from 3475 to 4863) for Rio de Janeiro. These percentages refer to the increase in death records that didn’t reference COVID-19. Thus, one can see a significant increase in the number of deaths during the epidemic period that attributed to causes that deviate from the expected pattern.

The discrepancy is clearly very large, in terms of percentage values, with respect to the reports on deaths due to diseases considered in this research and other causes, especially SARS, which reported an increase of around 5820% (from 8.04 to 476) in Manaus and 2880% (from 23.32 to 695) in Recife.

**Table 5:** Difference (∆) between real and predicted values.

Cities (epidemiological weeks)		∆ Deaths				∆ Total deaths
		Respiratory failure	Pneumonia	SARS^a^	Other causes	
**Belém (15th to 17th )**						
	Difference	88	90	178	125	481
	Increase (%)	127.24	60	1715.56	49.41	99.61
**Fortaleza (14th to 18th )**						
	Difference	69	52	157	248	526
	Increase (%)	52.69	23.47	815.20	27.33	41.17
**Manaus (13th to 19th)**						
	Difference	220	477	467	678	1842
	Increase (%)	611.11	196.12	5820.40	68.75	144.74
**Recife (14th to 19th)**						
	Difference	18	11	671	41	741
	Increase (%)	25	35.48	2880.27	9.13	128.92
**Rio de Janeiro (13th to 19th)**						
	Difference	319	483	391	194	1387
	Increase (%)	96.28	42.49	2284.8	9.7	39.96
**São Paulo (10th to 17th)**						
	Difference	851	301	274	339	1765
	Increase (%)	91.15	24.31	493.53	6.77	24.40

## Discussion

### Principal Findings

It is reasonable to assume that the values presented in Table 5 were incorrectly reported, concealing the actual number of deaths due to the pandemic. The reporting bias for COVID-19 (relating to respiratory diseases) may have occurred due to delays in releasing the results, lack of tests, or even errors in identifying the disease. It is important to stress that even other causes of deaths increased significantly during the pandemic period (eg, an increase of 68% [from 677.92 to 1664] in Manaus). This study attributes some of these deaths to COVID-19 as well.

Therefore, the extrapolated (period not covered in the historical series) values of the number of deaths were attributed to the underreporting of the pandemic. Table 6 shows the estimates of the percentage of underreporting of COVID-19–related deaths for each city compared to the official number of deaths up to May 21, 2020.

For the cities of this case study, an average underreporting of 40.7% is estimated for deaths related to COVID-19. The values vary between 25.9% to 62.7%, with emphasis on Manaus, which had the highest number of deaths underreported (62.7%), and Recife, with almost 50%. Fortaleza had the lowest number, with 25.9% of underreporting, in spite of its count being substantial.

**Table 6:** Underreported deaths due to coronavirus disease (COVID-19).

City	Population, N (PNAD^a^)	Extrapolated number of predicted deaths	Official number of deaths^b^	Total number of estimated deaths	Number of deaths per 1 million inhabitants	Underreported deaths (%)
Belém	1,492,745	481	952	1433	959.98	33.57
Fortaleza	2,669,342	526	1503	2029	760.11	25.92
Manaus	2,182,763	1842	1094	2936	1345.08	62.74
Recife	1,645,727	739	747	1486	902.94	49.73
Rio de Janeiro	6,718,903	1387	2376	3763	560.06	36.86
São Paulo	12,252,023	1765	3238	5003	408.34	35.28

^a^PNAD: Pesquisa Nacional por Amostra de Domicílios (National Household Sample Survey).

^b^As of May 21, 2020,

The National Household Sample Survey (Pesquisa Nacional por Amostra de Domicílios, PNAD) of the IBGE compiles data based on the socioeconomic characteristics of the Brazilian population [12]. By analyzing the number of deaths and population counts from the PNAD (Table 6), one can see the differences in underreporting and number of deaths per 1 million inhabitants for each city. The differences are also found in Table 5; there are several disagreements for underreporting bias for COVID-19. The differences may have occurred due to the distinct socioeconomic characteristics of each city, such as demographic density, HDI, population age group, access to health care, and number of intensive care unit (ICU) beds available, etc.

São Paulo, for example, ended up with the least number of deaths in terms of percentage (per population) and the least total difference (percentagewise) in deaths for the period not covered in the historical series (Table 5). Moreover, São Paulo has the highest HDI (0.8) in Brazil. It has a one of the highest numbers of ICU beds in the country—22.3 ICU beds per 100,000 inhabitants [27], which is much higher than necessary. On the other hand, Manaus, one of the most affected cities in Brazil, showed the highest difference in records for the extrapolated period not covered in the historical series (Table 5) and the highest number of deaths (population wise) as well as underreporting of deaths. Manaus has the lowest HDI (0.73) among the six capital cities and 9.63 ICU beds per 100,000 inhabitants, the smallest number among the considered cities.

In a recent study, EPICOVID19-BR, carried out by the Federal University of Pelotas (UFPel) [28], researchers interviewed and tested (for SARS-CoV-2) a group of people selected by lottery in the cities identified as the most affected in the country. The objective was to estimate the number of infectees for each city. The first stage considered 133 cities from all Brazilian states and took place between May 14-21, 2020. In this study, the authors reported the following percentage values of infection: Belém (15.10%), Fortaleza (8.7%), Manaus (12.5%), Recife (3.2%), Rio de Janeiro (2.2%), and São Paulo (3.1%).

In the context of EPICOVID19-BR, fatality rates were estimated using the total deaths predicted, along with the official figures of infections and the number of infections estimated by UFPel [28]. The discrepancy between the official number of the fatality rates—Belém (0.64%), Fortaleza (1.37%), Manaus (1.08%), Recife (2.82%), Rio de Janeiro (1.62%), and São Paulo (2.22%)—becomes evident as there is much difference between official figures and counts reported by EPICOVID19-BR. These rates ​​are compatible with those found in several studies [7,29,30]. Therefore, it is estimated that mortality values range from 0.64% (Belém) to 2.82% (Recife), and is much more reliable with respect to officially published counts. Emphasis must be given to the results presented by UFPel (CI 4.8%), which confirms the hypothesis that there is a substantial underreporting not only in the number of deaths but also and especially in the number of infections published by official government bodies.

Another relevant study, from Imperial College [7], estimated the COVID-19 impact in Brazilian states from February 25, 2020 to May 6, 2020, using a hierarchical Bayesian model. This model estimates the number of infections, deaths, and reproduction. These fatality rates are estimated to be much more optimistic than those from UFPel. The following fatality rates were calculated: Belém (Pará: 0.9%), Fortaleza (Ceará: 1.1%), Manaus (Amazon: 0.8%), Recife (Pernambuco: 1.1%), Rio de Janeiro (Rio de Janeiro: 0.8%), and São Paulo (São Paulo: 0.7%).

From the several fatality rates investigated (up to the time this study was conducted), and considering the main countries affected by the pandemic and number of predicted deaths in our research, it is possible to estimate the number of infected cases and consequently estimate the percentage of underreporting of infected cases. Table 7 presents estimations of the numbers of those that were infected in each city considering different fatality rates and also shows the estimated percentage of underreporting of infected cases.

Depending on how high or low the fatality ratio is, there is variation in the number of infected cases. For example, as seen in Table 7, the number of cases for São Paulo is estimated to be almost 76,000, considering the highest fatality ratio (Brazil, 6.6%), or approximately 715,000 when considering the lowest fatality ratio (Imperial College, 0.7%).

Based on these differing fatality rates, underreported infection numbers may be monumental. For example, underreporting of infected cases in Manaus (using the fatality ratio from the Imperial College study [7]) and Belém (using the fatality ratio from the EPICOVID19-BR study [28]) may reach 2880% and 2837%, respectively. Such scenarios show, in both the cities, a count that is 30 times the number of confirmed cases. For other capital cities, the numbers may be up to 11 (Recife), 12 (Fortaleza), 17 (São Paulo), and almost 25 times (Rio de Janeiro).

There were 739,503 confirmed cases and 38,406 official deaths, as of June 9, 2020 [9]. If we consider the average percentage of 40.7% for underreporting of deaths as shown in this study, Brazil would have around 64,746 deaths related to COVID-19. Considering the lowest and highest percentage of underreporting presented by the cities studied (Table 6), it would have around 51,846 (25.9%) and 103,071(62.7%) deaths, respectively, thus, estimating a much higher number of deaths than those officially reported.

**Table 7:** Estimated number of infection cases and percentage of cases underreported considering differing estimations in fatality rate.

Cities (predicted number of deaths)		Official count^a^	Fatality rate					
			UFPel^b^ [26]	Imperial College [7]	China (1.38%) [27]	Brazil (6.6%)	United States (6%)	Global (6.5%)
**Belém (n=1433)**		7675						
	Infections, n		225,404	159,222	103,841	21,712	23,883	22,046
	Underreported (%)		2837	1975	1253	183	211	187
**Fortaleza (n=2029)**		1864						
	Infections, n		232,233	184,455	147,029	30,742	33,817	31,215
	Underreported (%)		1146	889	689	65	81	67
**Manaus (n=2936)**		12,317						
	Infections, n		272,845	367,000	212,754	44,484	48,933	45,170
	Underreported (%)		2115	2880	1627	261	297	267
**Recife (n=1486)**		11,584						
	Infections, n		52,663	135,091	107,681	22,515	24,767	22,861
	Underreported (%)		355	1066	830	94	114	97
**Rio de Janeiro (n=3763)**		18,743						
	Infections, n		147,816	470,375	272,681	57,015	62,717	57,892
	Underreported (%)		689	2410	1355	204	235	209
**São Paulo (n=5003)**		41,451						
	Infections, n		379,813	714,714	362,536	75,803	83,383	76,969
	Underreported (%)		816	1624	775	83	101	86

^a^As of May 21, 2020.

^b^UFPel: Federal University of Pelotas.

Regarding the number of those infected by the pandemic, based on the value previously calculated for the number of total deaths (40.7%, 64,746 deaths), it can be inferred that Brazil’s count of infection ranges between 981,013 and 5,395,571 (considering respectively the highest and lowest lethality rate, 6.6% and 1.2%, respectively [7]). Hence, it is reasonable to assume that Brazil either is, or may become in the near future, the new epicenter of the COVID-19 pandemic, surpassing the United States, which of June 9, 2020, has the highest number of infected persons (n=1,933,560) [8].

When comparing both countries, the United States currently performs more tests for the disease than any other country in the world [31]. According to WorldoMeter [15], the United States has conducted 22,624,758 tests—70,799 tests per 1 million inhabitants. These numbers are well ahead of Brazil, which so far has conducted a total of 1,182,581 tests—5566 tests per 1 million inhabitants. Thus, with the testing coverage in the United States being much larger, the actual impact of the pandemic can be more realistically analyzed in that country and, therefore, in comparison to Brazil, more effective actions can be carried out to control the disease.

It is also worth considering the tendency to flatten the evolution curve of COVID-19, which represents the reduction in the number of daily new cases. We compared the evolution of weekly confirmed cases from United States and Brazil, up to June 9th. The reduction in the number of occurrences in the United States indicates that the curve is flattening. In contrast, the number of weekly confirmed cases in Brazil is still increasing. This ascending curve indicates that the pandemic is still growing, tending to surpass the official number of infected Americans in the near future when considering the official numbers. If we consider the highest lethality rates presented in this work, the actual number of infected Brazilian citizens would have already surpassed that of the United States.

### Conclusions

The significant rates of underreporting of deaths presented in our research indicate that the counts released by the official Brazilian internet portals are much lower than the actual numbers, making it impossible for the authorities to take more effective action. This is also confusing to citizens, who have demonstrated failure to comply with social isolation measures. Therefore, a public access portal is being developed in order to disseminate more realistic and reliable data on the pandemic, in order to undo the contradictions of official data, guide the population, formulate policies, and estimate the R factor more efficiently.

Our results suggest a growing pandemic and reveal a wide heterogeneity in the outbreak of the epidemic in the cities considered in this case study, suggesting a greater number of underreporting in deaths and infected cases in some cities. This demonstrates differing levels of the outbreak stage, more advanced in some cities compared to others. However, in no city do the results indicate that herd immunity is close to being achieved. In addition, the underreporting of deaths is not stationary over time and may increase during the pandemic period.

The number of deaths due to SARS was considerably higher than the expected number for all six cities, indicating that a large number of deaths related to COVID-19 were possibly mistakenly recorded as SARS. It is assumed that this is due to lack of confirmation and delays in testing or confusion in diagnosis, since COVID-19 is a new disease. Furthermore, delays in disclosing test results also impact the effect and reach of the pandemic. Therefore, it is of paramount importance to increase testing in order to reduce underreporting and encourage rapid dissemination of test results to allow for a closer view of the real COVID-19 situation in Brazil.

## Abbreviations

COVID-19: coronavirus disease
DATASUS: Department of Informatics of the Unified Healthcare System
GDP: gross domestic product
HDI: Human Development Index
IBGE: Brazilian Institute of Geography and Statistics
ICD-10: International Statistical Classification of Diseases and Related Health Problems–10th Revision
PNAD: Pesquisa Nacional por Amostra de Domicílios (National Household Sample Survey)
R: reproduction number
RT-PCR: reverse transcription polymerase chain reaction
SARS: severe acute respiratory syndrome
SARS-CoV: severe acute respiratory syndrome coronavirus
SARS-CoV-2: severe acute respiratory syndrome coronavirus 2
SIM: Mortality Information System
SUS: Sistema Único de Saúde (Unified Healthcare System)
UFPel: Federal University of Pelotas
WHO: World Health Organization
